# Risk Intelligence as a Resource in Career Transition: The Role of College Satisfaction on the Visions about Future Jobs

**DOI:** 10.3390/ejihpe11030077

**Published:** 2021-09-07

**Authors:** Ernesto Lodi, Andrea Zammitti, Paola Magnano

**Affiliations:** 1 Department of Humanities and Social Sciences, University of Sassari, Via Roma 151, 07100 Sassari, Italy; 2 Department of Science of Education, University of Catania, Via Biblioteca 4, 95124 Catania, Italy; andrea.zammitti@phd.unict.it; 3 Faculty of Human and Social Sciences, Kore University, Cittadella Universitaria, 94100 Enna, Italy; paola.magnano@unikore.it

**Keywords:** college satisfaction, career path, risk intelligence, visions about future job, college students, university wellbeing, career transitions, future expectations about work

## Abstract

(1) Background: University transition is a critical step in career construction due to the uncertainty and unpredictability of socioeconomic conditions; these conditions compel people to manage a greater quantity of perceived risks associated with their career projects than in the past, and to face unexpected situations that could compromise their quality of life in educational and work contexts. After all, experiencing well-being during the university path can undoubtedly affect the visions of one’s future work, especially when a transition period is nearby. The present study aimed to explore the role of subjective risk intelligence in expectations about future work, analyzing the potential mediational role of academic satisfaction in this relationship. (2) Methods: A longitudinal study was carried out on 352 Italian university students at the end of the degree course. We used the following measures: in T1, Subjective risk intelligence scale, College Satisfaction scale; in T2, three items assessing the expectations about future work. (3) Results: The main findings showed that subjective risk intelligence has both direct and indirect effects (through the mediation of college satisfaction) on the expectations about future work. (4) Conclusions: The ability to manage risks, also through the contribution of domain-specific satisfaction, can lead to positive expectations toward one’s future work. This could increase the likelihood to perform career-related behaviors in a more proactive way if people have high risk management skills and high levels of academic satisfaction with their university path during transition.

## 1. Introduction

University students who approach the completion of their degree can experience disparate feelings and attitudes by imagining their working future (from the most positive to the most negative expectations), seeing the transition phase from university to the world of work being real. Having positive expectations toward one’s future work can undoubtedly affect the actions that will then be concretely implemented daily at the end of the degree program. The aim of this article was to discuss how the well-being experienced at university level can act as a support for positive visions of future work, considering some fundamental variables during the transition phases, for example, the ability to cope with perceived risks and the emotions associated with them. The risk perceptions are indeed derived (and increased) from a labor market that has been totally and rapidly changed, and these transformations have deeply influenced the development of career paths [1]. These changes and transformations had already had a profound impact on the world of work (for example, by reducing the availability and quality of jobs, and the well-being of workers), and the most recent studies showed that these effects have now been amplified even more by the pandemic situation linked to COVID-19, particularly affecting the most vulnerable categories [2,3,4]. For university students, recent studies have underlined that the pandemic situation negatively affected academic achievement, increased the risk to the delay in the degree, and the negative expectations about future work. Therefore “students face an increasingly uncertain environment, where financial and health shocks (for example, lack of resources to complete their studies or fear of becoming seriously sick), along with the transition to online learning, may have affected their academic performance, educational plans, current labor market participation, and expectations about future employment” [5] (p. 2).

One of the most principal approaches in the field of the construction of contemporary career paths can be identified in the Life Design paradigm, a perspective based on the epistemology of social constructionism [6]. It emphasizes the continuous evolution of the person and society that is increasingly characterized by complexity and great changeability. Savickas [7], identifying unpredictability as the prominent characteristic of careers and work in the third millennium, underlined that the individuals should be enabled to respond adaptively to continuous changes, dealing effectively with instability and risks that characterize the world of work. A successful career transition can be favored by individuals’ psychological resources applied to different contextual conditions related to the transition and on the full use of their psychological resources [8].

The abilities to cope with the uncertainty and risks associated with the future and feel high levels of well-being in the educational contexts that people cross during life paths are the two main variables in our article. Therefore, as the Life Design paradigm narrates today’s nonlinearity and complexity of career paths, the ability to manage the risks associated with those paths can have a great impact on how people imagine their professional future. Satisfaction with the university career undertaken could contribute to these effects. Academic satisfaction is closely correlated with success and progress toward one’s academic goals. Responding effectively to educational challenges, adapting well to university life, producing effort and being motivated, and achieving learning goals are variables linked to the levels of well-being experienced in college, and this can also affect the subsequent impact on post-college performance [9,10,11]. The way in which people live through university, the degree of positive adaptation, albeit with the expected difficulties, and the feeling of competence in being an effective student and in contributing to academic satisfaction will, in turn, lead the students to imagine future positive or negative scenarios. Imagining positive scenarios, characterized by a satisfactory job, consistent with the professional interests, and not in too long a time, can affect the behavioral intention and the subsequent actions [12] that they will implement in active research of a job; this dynamic will lead students in a university-to-work transition to be proactive and not passive or discouraged from the current fluid situation of the job market.

### 1.1. Risk Intelligence and Career

Numerous factors influence academic success, and some authors [13] have ranked predictors of academic achievement by personality traits or dimensions, motivation, self-regulation, and psychosocial and relational factors. The interest in the study of nonintellectual factors derives from the fact that they can be improved [14,15], and this represents an important working area for career counseling activities. Some dimensions have been extensively studied in their relationship to academic achievement: self-efficacy positively influences career exploration [16] and academic achievement [17]; academic achievement is positively correlated with self-esteem [18,19,20,21,22]; Creed, Patton, and Bartrum [23] showed that optimism is related to high levels of career planning, exploration, decision-making confidence, and career-related goals; hope correlates positively with career planning [24] and vocational identity [25,26]. There are still many dimensions related to academic success, but an exhaustive list of these was not the goal of this paper. It is important to reflect, however, on the fact that the ability to make career transitions that allow people to be successful in the job may depend in part on the use of these resources [8].

A dimension that has recently become part of the career counseling field is risk intelligence. Craparo and colleagues [27] defined subjective risk intelligence “as the capacity of a person to effectively assess the pros and cons of a decision in situations in which not all outcomes are totally expected” [27] (p. 968). As career changes are classified as risky situations [28], risk intelligence can support people to effectively assess risks and feel adept at managing the uncertainty that can arise from their choices [27]. The literature on the role of subjective risk intelligence in career construction has not yet been deepened, as the construct has been recently conceptualized as an individual dimension. In fact, the concept of risk intelligence comes from the managerial field and refers to certain kinds of behavior adopted in business organizations. The most representative conceptualizations of this construct were provided by Evans [29], who defined risk intelligence as the ability to accurately estimate probabilities; by Apgar [30], who talked about the ability of an individual or an organization to effectively evaluate risks; and by Funston and Wagner [31], who identified risk intelligence as the capacity to distinguish between risks that must be avoided for the sake of survival, and risks that must be taken to gain a competitive advantage.

According to the theorization of Craparo and colleagues [27], individuals with high levels of subjective risk intelligence can evaluate uncertainty as an opportunity rather than a danger, they have creative behavior, they can manage stress, and they have high levels of self-confidence and of ability to make decisions. The construct, therefore, is formed by these four dimensions: (1) imaginative capability, which refers to the production of new ideas [32]; (2) problem-solving self-efficacy, with self-confidence and the ability to make decisions; (3) emotional stress management, which measures the capacity to handle emotional responses in stressful situations; and (4) positive attitude toward uncertainty, which refers to the ability to perceive uncertainty as an opportunity rather than a threat.

In a recent study on the relationship between risk intelligence and personal values [33], it emerged that this construct correlates positively with the values of openness to change, self-transcendence, and self-enhancement; furthermore, values of openness to change and self-transcendence can help people manage uncertainty through risk intelligence. In previous studies, it emerged that risk intelligence positively correlates with adaptive coping strategies and emotional intelligence [27]. Furthermore, risk intelligence is a psychological resource that can be involved in career transition processes. Risk intelligence is not an innate talent, and career counseling experiences can help enhance this important psychological resource.

### 1.2. Academic Satisfaction

Academic satisfaction can be defined as the enjoyment of one’s experiences related to the student role [34] and involves the achievement of academic objectives and aspirations [35]. When evaluating academic satisfaction, many authors agree on its multidimensional nature, as it involves multiple aspects of the personal academic experience: the behavioral level through the results, the academic performance, and the attitudes toward study; the relational level associated with the quality of the relationship with colleagues and professors; the level related to the construction of one’s career regarding the perceived usefulness of the course attended to carry out the desired job (or jobs) [36]. Therefore, the consistency between the knowledge and the competences acquired and their usefulness for the future work is a core aspect of the students’ academic satisfaction [37].

In the socio-cognitive framework, academic satisfaction can be influenced by several domain-specific variables, such as self-efficacy in an academic context, goal-directed activity, and environmental supports for the academic aims; moreover, it is related to the overall satisfaction with life [38] and psychological well-being [39]. Academic satisfaction is also related to career development variables: it is influenced by the perception of congruence between one’s own professional interests and the specific college course chosen [40,41]; it is negatively correlated with career choice anxiety and indecision and positively correlated with career decision self-efficacy [42].

As for our specific study, we assumed that students’ satisfaction can improve the students’ expectation of success [43]. To support this assumption, the recent literature on positive psychology applied to career choices has shown that satisfaction with one’s educational context is related with optimism, hope, the ability to imagine positive future scenarios, and the orientation toward the future. Most of the studies in the literature on these issues have used mainly students’ life satisfaction, rather than a strictly domain-specific measure. For example, Cabras and Mondo [44] showed that future orientation is related to students’ life satisfaction. Therefore, other recent studies have highlighted that the levels of optimism are related to greater levels of academic satisfaction [45], and to students’ life satisfaction and psychological well-being in samples of college students [46,47]. Theory and research about the development of career paths underline that domain-specific satisfaction in a valued context (i.e., for the school and the university attended) is related to the use and improvement of positive resources and planning skills in career projects; in fact, the positive experience in the educational context derives from the opportunity to acquire and improve the skills, the professional interests, and the competencies, which, in turn, will support future transitions [48]. The academic satisfaction with university courses can predict the level of future positive variables linked to the work context (e.g., involvement in work), as it is related to the career competencies and subjective career achievement [1,49,50]. Finally, a hopeful attitude toward the future is related to college students’ mental health, psychological and physical health, and positive life outcomes at the various educational levels [51,52,53].

Given this scenario, academic satisfaction could mediate the effect of risk intelligence on future expectations, precisely because they are formulated in a period characterized by great uncertainty.

### 1.3. Purpose of the Study

Applying the most recent development of the theory of planned behavior [11], subjective risk intelligence—reflecting a belief system about oneself—would lead to positive attitudinal and affective states, such as academic satisfaction; these positive attitudinal states, in turn, activate expectations about future work that would presumably lead to behavioral changes, such as job search. Following this reasoning, we hypothesize that the dimensions of subjective risk intelligence, which are cognitive and emotional constructs that support individuals in managing risky and uncertain situations, have an effect on the expectations about finding a job in a short period of time, which is consistent with the competences acquired during the degree course, and is satisfying for at least one important aspect; moreover, we hypothesize that this effect could be mediated by two areas of the satisfaction for the degree course attended: satisfaction about the degree course chosen and the satisfaction about the utility of the degree course for future careers. Thus, we hypothesize that the dimensions of subjective risk intelligence have both a direct and indirect effect on the expectations to find consistent work, to find work in a short time, and to find satisfying work; the indirect effects are hypothesized to be mediated by the choice satisfaction and the utility satisfaction.

## 2. Materials and Methods

### 2.1. Research Design

The study was conducted through a longitudinal design: in the first step (T1), the subjective risk intelligence and the academic satisfaction were detected; after one year from the T1, the respondents—who had completed their degree course—were asked to respond to a survey, comprising, among others, three questions exploring expectations about future work (T2).

### 2.2. Participants and Procedure

The study involved 352 university students (M = 100; F = 252), aged from 21 to 29 years (M = 24.09; SD = 4.18), attending the last year of their degree course, who completed both the steps, T1 and T2. At T1, 513 students filled in the research protocol; thus, the response rate between T1 and T2 was 68.62%. The different degree courses attended were as follows: socio-psychological and educational area = 140 (39.8%); physical and sports activities = 71 (20.2%); humanistic degree courses = 55 (15.6%); economic, politic, and juridic degree courses = 30 (8.5%); health professions courses = 26 (7.4%); the remaining part (30, 8.5%) was distributed among tourism, biologic and natural sciences, and engineering. The respondents attended different universities in the north, center, and south of Italy.

### 2.3. Procedure

After having expressed their informed consent to the use of data, the participants completed an online survey, being free to abandon the compilation at any time. The respondents were required to provide a personal code, composed of the birth year, the first two letters of the last name, and the first two letters of the first name. This code, matched with other demographic information, allowed us to check any eventual double compilation. Providing their email addresses, the respondents allowed the researchers to contact them again for the T2 administration, which followed the same rules about privacy management. In T2, too, the participants expressed their informed consent. The survey was approved by the ethical committee of the universities involved and followed the rules of the Italian Association of Psychology for the psychological research.

### 2.4. Measures

#### 2.4.1. Subjective Risk Intelligence Scale

The Subjective Risk Intelligence Scale (SRIS, [27]) has been created and validated for the Italian population and is composed of twenty-one items; the items describe behaviors or moods, and the respondents answered using a five-point Likert-type scale from 1 (totally disagree) to 5 (totally agree). The items are grouped in four dimensions: (1) emotional stress management (sample item: “when I feel fearful about something, I have difficulty concentrating on everything” Cronbach’s alpha = 0.76) measures the capacity to modulate emotional responses in stressful situations; (2) attitude toward uncertainty (sample item: “the uncertainty about possible developments of a situation paralyzes me” Cronbach’s alpha = 0.84) refers to the ability to perceive uncertainty as an opportunity rather than a threat, attributing positive significance to it; (3) imaginative capability (sample item: “to be able to create new procedures, I think for myself instead of following procedures established by others”; Cronbach’s alpha = 0.81) refers to the generation of novel and potentially useful ideas [32], emphasizing the attributes of initiative-taking and originality; this dimension includes an individual’s ability to explore the unknown [54]; (4) problem-solving self-efficacy (sample item: “I feel able to make decisions even when I don’t have all the information” Cronbach’s alpha = 0.75) comprises both self-confidence and beliefs in one’s capacity to handle situations, including the ability to make decisions.

#### 2.4.2. College Satisfaction Scale

The College Satisfaction Scale (C-Sat Scale; [36]) measures the degree of college satisfaction in a multidimensional perspective; it has been created and validated for the Italian population and is composed of 20 items grouped in four areas of satisfaction. Consistently with the purpose of the study, we selected the following scales: the appropriateness of the student’s choice (choice), which is how satisfied the students feel about their chosen degree program (sample item: “I am satisfied … For choosing this academic path”; Cronbach’s alpha = 0.85); the usefulness for the future career (utility), which is how satisfied the students feel about their degree course in terms of the utility of the competences developed for their future work, the positive effect on their future career, and improvement in realizing job aspirations (sample item: “I am satisfied … Because my studies will be useful for finding future employment”; Cronbach’s alpha = 0.91) [9].

#### 2.4.3. Expectations about Work

The expectations about work were assessed using three ad hoc items, with a 10-point Likert scale, from 1 (not at all probable) to 10 (extremely probable). The items were: how likely do you think, after graduation … (1) to find a job that is consistent with your studies (consistent work); (2) to find a job within 3 years of completing your studies (work in a short time); (3) to find a job that satisfies you for at least one aspect that you consider important (e.g., economic, type of activities carried out, etc.) (satisfying work).

### 2.5. Data Analysis

Data analysis was conducted using the following strategy: descriptive statistics and correlations were calculated through SPSS 25.0. After having verified the correlations between the variables included in the study, we tested the mediational hypotheses. The mediational analysis was conducted using JAMOVI 1.6.23 [55], reporting the significance of the indirect effects obtained through the bootstrapping method with 5000 repetitions, with a confidence interval (CI) of 95%. We reported the standardized βs, to indicate the intensity of the effect, and the confidence intervals (CIs) 95%, which indicate the significance of the effect with a 5% of probability of error (CIs that do not comprise 0 are significant).

## 3. Results

### 3.1. Descriptive Statistics and Correlations

We analyzed the relationships between the variables of the study using the Pearson’s r coefficient. We found significant and strong correlations between the two dimensions of academic satisfaction (choice and utility) and the three items detecting the expectations about work; significant but weaker relationships are shown between three out of four dimensions of subjective risk intelligence and the choice and utility satisfaction; more specifically, imaginative capability and problem-solving self-efficacy are positively related to the satisfaction dimensions; attitude toward uncertainty is negatively related to both satisfaction dimensions; stress management is not related to any of them. Moreover, the same pattern is shown by the relationships between the dimensions of subjective risk intelligence and the three items on the expectation about work: imaginative capability and problem-solving self-efficacy are significantly and positively related to the expectations about work; attitude toward uncertainty is negatively related to two out of three items on expectations about work (consistent work and satisfying work); any significant relationship was not found between stress management and the expectations about work. The descriptive statistics and the Pearson’s coefficients are reported in Table 1.

### 3.2. Mediation Analysis

**Figure 1:**
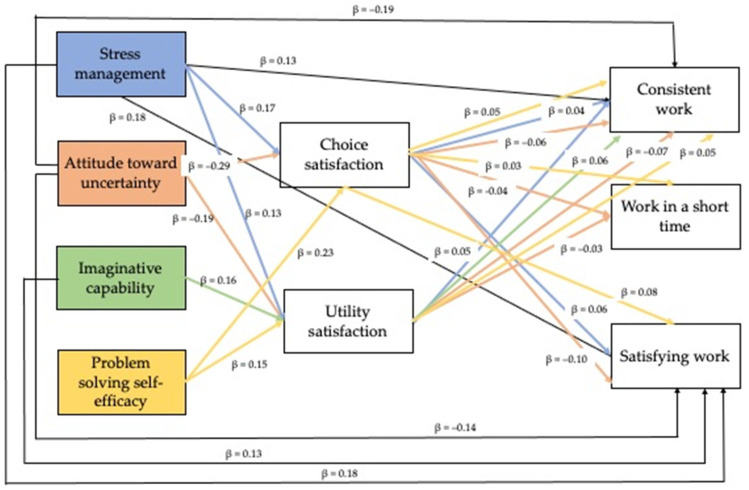
The mediation model.

The mediational hypothesis was tested using the bootstrapping method to verify the significance of the indirect effects. Stress management and attitude toward uncertainty have both a direct and indirect effect—mediated by choice satisfaction—on consistent work and satisfying work; imaginative capability has a direct effect on satisfying work; problem-solving self-efficacy does not have any direct effect on the expectations about work. Moreover, utility satisfaction plays a mediational role in the relationship between stress management and attitude toward uncertainty with consistent work; the relationships between attitude toward uncertainty and problem-solving self-efficacy with work in a short time are fully mediated by choice satisfaction; both choice and utility satisfaction fully mediate the relationship between problem-solving self-efficacy and consistent work; the relationship between problem-solving self-efficacy and satisfying work is fully mediated by choice satisfaction, too. Finally, choice satisfaction fully mediates the relationship between imaginative capability and consistent work. Figure 1 presents the mediation model.

Table 2 presents the results of the mediations, reporting the standardized βs. The results showed that stress management has a direct relationship on consistent work (DE = 0.133, *p* = 0.017, CI = 0.02, 0.194) and satisfying work (DE = 0.183, *p* = 0.001, CI = 0.054, 0.214); moreover, the paths from stress management to choice satisfaction (β = 0.17) and utility satisfaction (β = 0.13) are significant, as well as the path from choice satisfaction to consistent work (IE = 0.035, *p* = 0.018, CI = 0.005, 0.050) and the path from utility satisfaction to consistent work (IE = 0.047, *p* = 0.035, CI = 0.003, 0.072). Thus, the relationship between stress management with consistent work is partially mediated by both the mediators, choice and utility satisfaction, whereas the relationship with satisfying work is partially mediated by choice satisfaction (IE = 0.058, *p* = 0.008, CI = 0.011, 0.074).

Attitude toward uncertainty has a direct relationship on consistent work (DE = −0.186, *p* = 0.001, CI = −0.147, −0.036) and satisfying work (DE = −0.140, *p* = 0.015, CI = −0.114, −0.012); moreover, the paths from attitude toward uncertainty to choice satisfaction (β = −0.29) and utility satisfaction (β = −0.19) are significant, as well as the path from choice satisfaction to consistent work (IE = −0.059, *p* = 0.001, CI = −0.047, −0.011) and the path from utility satisfaction to consistent work (IE = −0.069, *p* = 0.003, CI = −0.056, −0.012). Thus, the relationship between attitude toward uncertainty and consistent work is partially mediated by both the mediators, choice and utility satisfaction, whereas the relationship between attitude toward uncertainty with work in a short time is fully mediated by both the mediators, choice (IE = −0.042, *p* = 0.014, CI = −0.039, −0.004) and utility (IE = −0.034, *p* = 0.02, CI = −0.032, −0.003) satisfaction. Then, the relationship with satisfying work is fully mediated by choice satisfaction (IE = −0.100, *p* < 0.001, CI = −0.067, −0.023).

Imaginative capability shows only a direct relationship on satisfying work (DE = 0.15, *p* = 0.002, CI = 0.012–0.052); moreover, utility satisfaction fully mediates the relationship with this dimension and consistent work (IE = 0.056, *p*, CI = 0.001, 0.051).

Finally, the relationship between the three items on the expectations about work and problem-solving self-efficacy is fully mediated by choice satisfaction (consistent work: IE = 0.047, *p* = 0.011, CI = 0.006, 0.046; work in a short time: IE = 0.033, *p* = 0.039, CI = 0.022, 0.038; satisfying work: IE = 0.079, *p*, CI = 0.014, 0.068); utility satisfaction further mediates for consistent work (IE = 0.054, *p* = 0.045, CI = 0.027, 0.059).

## 4. Discussion

The results of the study show that the four dimensions of subjective risk intelligence are related to the expectations about future work, both directly and indirectly, through the mediation of two aspects of academic satisfaction—satisfaction for the degree course chosen and for its perceived usefulness for the future professional career. More specifically, we discuss the single patterns.

First, students able to manage emotional stress are more likely expected to find a job that is satisfying and consistent with their knowledge and competences. These expectations are favored by the perception of satisfaction about the choice and the utility of the degree course. The ability to manage emotions, maximizing positive ones and minimizing negative ones, can affect domain-specific levels of well-being. This is true if we consider well-being in the hedonic perspective, which include positive and negative effects as main components [56]; moreover, these results are in line with both Seligman’s PERMA model [57], which considers the role of positive emotions among the five main elements of well-being, and the socio-cognitive model of well-being, in which the levels of positive and negative emotions influence the experience of well-being [58]. Being competent in managing one’s emotions, despite the perception of risks, and in a critical phase of transition, can increase the levels of students’ well-being, which, in turn, can lead to the vision of a future that is positive regarding the expectations about the future.

Secondly, the propensity toward uncertainty has surprisingly shown a negative relationship, both in the direct and indirect effects, with the three expectations about work. The satisfaction about choice mediates the relationship with all the three expectations, to find a job in a short time, consistent with the degree course and satisfying; the satisfaction about utility mediates the relationship with the expectation to find a consistent job and in a short time. There are no results in the literature review conducted that can support this finding. The aspect that solicits our reflection is that the positive attitude toward uncertainty perhaps leads people to not perceive as totally satisfactory a path that defines themselves in a “stable” way as they desire an uncertain, but more stimulating, future. One of the hypotheses that we could test in the future is whether this aspect of risk intelligence could be linked to a sort of recklessness that also pushes the individuals to mull over the choices made and to be dissatisfied with the things that are learned. Could people who prefer uncertainty be more likely to think that the alternatives they left behind during their career decisions could be better than the choice they made? This could therefore lead to thinking of a more uncertain and unstable future, almost because of a kind of cognitive coherence.

Third, imaginative capability and initiative-taking, through the support of satisfaction about the usefulness of the degree course, help students in finding a job that is consistent with their professional interests. The result concerning the significant effect of the imaginative capacity on satisfaction with professional usefulness in view of a future job seems to be coherent, assuming the imaginative capacity as an orientation to the future rather than a reflection on the past. Moreover, according to our point of view, the relationship between imaginative capability and the production of positive future scenarios is very consistent with previous literature. If we consider Snyder’s definition of hope, it emphasizes that hope is the ability to produce multiple paths to achieve the desired goals [59]. As Marques et al. stated, “the production of several pathways is important when encountering impediments (…) pathways thinking refer to a person’s perceived ability to generate workable routes to desired goals” [52] (p. 140–141). This process led people to motivate themselves using those paths and mental goals, and in this way, hope is capable of influencing human behavior.

Finally, problem-solving self-efficacy helps students that are satisfied about the choice of their degree course to find a job in a short time, which is consistent with their interests and satisfying; the perception of the usefulness of the degree course, then, helps self-confident students in finding a job consistent with their interests. This result seems in line with the large amount of research produced in the field of socio-cognitive career theory [60], which conceptualizes the educational and professional contexts as areas closely interrelated and which has frequently shown the link between self-efficacy and domain-specific satisfaction (school, university, and work satisfaction and expectations of results [9,38,39,61]). Even when self-efficacy is applied generically to problem-solving, our results confirm the links mentioned above: greater level of self-efficacy affect positively the satisfaction for the present and the future career paths, helping people to imagine positive work scenarios. Bandura [62] already indicated the capacity of symbolization, anticipation, self-reflection, and self-regulation of the human mind, and probably, the more effective students feel in problem-solving, the more they can narrate themselves as competent in the actions that they will implement as the most appropriate in searching for a future job after university and the probable related problems that need to be solved. As our results show, this takes place through the mediation of domain-specific satisfaction.

## 5. Conclusions

This study showed how, during the university-to-work transition, risk intelligence and academic satisfaction are core variables in building future positive expectations regarding future work. This effect was analyzed within the paradigm of Life Design and the theory of planned behavior, considering the importance of well-being in the current perspectives of positive psychology. The construction of career paths and their development in the 21st century requires the ability to cope with risks and complex situations, and these skills can foster the perception of academic satisfaction, which, in turn, can increase the ability to build a future full of hope and optimism, sources of motivation for proactive behaviors related to job search and successful transitions.

The pandemic situation has undoubtedly increased the requests for psychological support, often without adequate specific training on dealing with crisis situations and without adequate economic and financial resources to cope with them [63]. Universities should therefore focus on strengthening counseling and psychological support services for careers and, at the same time, should focus on training their human resources; the Life Design approach offers a theoretical and methodological framework to plan and implement such trainings (for example, by increasing the skills in supporting risk management and career adaptability). These skills are extremely useful, especially when the career counsellor has to support the students’ difficulties as outcomes of the pandemic situation, i.e., the vulnerability of people in building dignified, satisfying, and successful career paths. University career counselors and professionals can use the results of this research to act in a preventive perspective on strengthening the ability to manage risks, a fundamental skill in today’s socio-economic situation. Enhancing this ability and, at the same time, increasing the satisfaction that students experience for the chosen college path, can help them to face the current world of work and prevent the possible negative effects of discomfort that can be experienced during the transitions. Psychoeducational training aimed at the improvement of the dimensions related to risk intelligence could be useful to support the students in developing the skills involved in facing effectively the critical stages of their careers. Creating “permanent” career guidance projects over the years could help to monitor and consolidate student satisfaction with the chosen path and its usefulness for future work, in a preventive way. Creating positive and supportive contexts can mitigate the effects of uncertainty and increase people’s ability to imagine a positive future while building their career path. Future research within the Life Design paradigm could be focused on building and validating training courses on risk intelligence in the wake of those already existing on career adaptability. Finally, evaluating the dimensions of our study, career guidance professionals can identify the points of strength or weakness of students in this field (especially when they are approaching the degree) and implement specific interventions to support a successful career transition, for example, helping students to manage the risk and to avoid the decrease in levels of well-being considering the difficulties to be faced and the fears related to the future job.

Future research could aim to clarify the negative effect that has been shown for the positive attitude toward uncertainty with respect to academic satisfaction and the construction of positive and well-defined scenarios related to future work. Future studies could use, for example, additional variables related to personality traits or the configuration of professional values (for example, those related to the challenges of the orientation and search for risk and unpredictability of work). Moreover, a specific research line could be dedicated to the construction and validation of the psychoeducational training to improve risk intelligence.

Our study and its results must also be analyzed in light of some limitations. The first is on a theoretical level, as there are not enough studies on the relationship between risk intelligence and domain-specific satisfaction measures. A further limitation is the use of self-report tools and the convenience sampling method with all associated biases that may have influenced our results; due to the longitudinal design of the research, a small number of participants were involved in the two stages. Finally, a longer longitudinal study would allow the evaluation of the actual outcomes both in the behaviors that students put into practice in the job search and in the working field found, such as evaluating job satisfaction or job engagement. This would have allowed us to arrive at more solid results, corroborating the hypotheses we formulated in a more precise way.

## Figures and Tables

**Table 1:** Descriptive statistics and correlations between the dimensions of the study (Pearson’s r).

	M	SD	1	2	3	4	5	6	7	8	9
1. Stress management	2.42	0.95									
2. Attitude toward uncertainty	2.78	0.92	0.52 ***								
3. Imaginative capability	3.56	0.69	−0.08	−0.08							
4. Problem-solving self-efficacy	3.55	0.68	−0.09	−0.01	0.71 ***						
5. Choice satisfaction	3.47	3.63	−0.01	−0.21 **	0.23 ***	0.26 ***					
6. Utility satisfaction	3.21	3.59	0.004	−0.14 **	0.27 ***	0.25 ***	0.73 ***				
7. Consistent work	7.06	2.33	0.03	−0.20 **	0.19 ***	0.16 **	0.46 ***	0.49 ***			
8. Work in a short time	7.28	2.38	0.06	−0.02	0.20 ***	0.19 ***	0.30 ***	0.32 ***	0.53 ***		
9. Satisfying work	7.21	2.11	0.09	−0.14 *	0.29 ***	0.27 ***	0.47 ***	0.40 ***	0.64 ***	0.56 ***	

* *p* < 0.05; ** *p* > 0.01; *** *p* < 0.001

**Table 2:** Effects of the dimensions of subjective risk intelligence on expectations about work through choice and utility satisfaction (standardized β).

Paths	Indirect Effect (IE)		Direct Effect (DE)		Total Effect	
	β	C.I. 95%	β	C.I. 95%	β	C.I. 95%
Stress management–Choice satisfaction–Consistent work	0.035	0.005, 0.050	0.133	0.020, 0.194	0.195	0.064, 0.255
Attitude uncertainty–Choice satisfaction–Consistent work	−0.059	−0.047, −0.011	−0.186	−0.147, −0.036	−0.289	−0.205, −0.087
Imaginative capability–Choice satisfaction–Consistent work	0.012	−0.008, 0.019	0.046	−0.039, 0.082	0.110	−0.015, 0.121
Problem-solving self-efficacy–Choice satisfaction–Consistent work	0.047	0.006, 0.046	0.026	−0.061, 0.090	0.097	−0.025, 0.014
Stress management–Utility satisfaction–Consistent work	0.047	0.003, 0.072	0.133	0.02, 0.194	0.195	0.064, 0.255
Attitude uncertainty–Utility satisfaction–Consistent work	−0.069	−0.056, −0.012	−0.186	−0.147, −0.036	−0.289	−0.205, −0.087
Imaginative capability–Utility satisfaction–Consistent work	0.056	0.001, 0.051	0.046	−0.039, 0.082	0.110	−0.015, 0.121
Problem-solving self-efficacy–Utility satisfaction–Consistent work	0.054	0.027, 0.059	0.026	−0.061, 0.090	0.097	−0.025, 0.014
Stress management–Choice satisfaction–Work in a short time	0.025	0.012, 0.040	0.070	−0.042, 0.157	0.116	−0.003, 0.198
Attitude uncertainty–Choice satisfaction–Work in a short time	−0.042	−0.039, −0.004	0.004	−0.061, 0.066	−0.071	−0.099, 0.025
Imaginative capability–Choice satisfaction–Work in a short time	0.008	−0.006, 0.014	0.087	−0.027, 0.111	0.121	−0.012, 0.131
Problem-solving self-efficacy–Choice satisfaction– Work in a short time	0.033	0.022, 0.038	0.056	−0.054, 0.0118	0.115	−0.018, 0.152
Stress management–Utility satisfaction–Work in a short time	0.023	−0.001, 0.040	0.070	−0.042, 0.157	0.116	−0.003, 0.198
Attitude uncertainty–Utility satisfaction–Work in a short time	−0.034	−0.032, −0.003	0.004	−0.061, 0.066	−0.071	−0.099, 0.025
Imaginative capability–Utility satisfaction–Work in a short time	0.028	−0.001, 0.028	0.087	−0.027, 0.111	0.121	−0.012, 0.131
Problem-solving self-efficacy–Utility satisfaction– Work in a short time	0.027	−0.002, 0.032	0.056	−0.054, 0.0118	0.115	−0.018, 0.152
Stress management–Choice satisfaction–Satisfying work	0.058	0.011, 0.074	0.183	0.054, 0.214	0.243	0.096, 0.265
Attitude uncertainty–Choice satisfaction–Satisfying work	−0.100	−0.067, −0.023	−0.140	−0.114, −0.012	−0.247	−0.165, −0.061
Imaginative capability–Choice satisfaction–Satisfying work	0.020	−0.012, 0.029	0.130	0.085, 0.111	0.163	0.011, 0.131
Problem-solving self-efficacy–Choice satisfaction– Satisfying work	0.079	0.014, 0.068	0.101	−0.017, 0.120	0.179	0.022, 0.165
Stress management–Utility satisfaction–Satisfying work	0.013	−0.003, 0.022	0.183	0.054, 0.214	0.243	0.096, 0.265
Attitude uncertainty–Utility satisfaction–Satisfying work	−0.019	−0.018, 0.001	−0.140	−0.114, −0.012	−0.247	−0.165, −0.061
Imaginative capability–Utility satisfaction–Satisfying work	0.015	−0.002, 0.015	0.130	0.085, 0.111	0.163	0.011, 0.131
Problem-solving self-efficacy–Utility satisfaction– Satisfying work	0.015	−0.003, 0.018	0.101	−0.017, 0.120	0.179	0.022, 0.165

## Data Availability

The dataset is available upon request to the corresponding author.

## References

[1] Lodi E., Zammiti A., Magnano P., Patrizi P., Santisi G. (2020). Italian Adaption of Self-Perceived Employability Scale: Psychometric properties and relations with the Career Adaptability and Well-being. Behav. Sci..

[2] Blustein D.L., Duffy R., Ferreira J.A., Cohen-Scali V., Cinamon R.G., Allan B.A. (2020). Unemployment in the time of COVID-19: A research agenda. J. Vocat. Behav..

[3] Di Fabio A., Svicher A. (2021). The Psychology of Sustainability and Sustainable Development: Advancing Decent Work, Inclusivity, and Positive Strengths-based Primary Preventive Interventions for Vulnerable Workers. Frontiers.

[4] Kniffin K.M., Narayanan J., Anseel F., Antonakis J., Ashford S.P., Bakker A.B., Bamberger P., Bapuji H., Bhave D.P., Choi V.K. (2021). COVID-19 and the workplace: Implications, issues, and insights for future research and action. Am. Psychol..

[5] Aucejo E.M., French J., Araya M.P.U., Zafar B. (2020). The impact of COVID-19 on student experiences and expectations: Evidence from a survey. J. Public Econ..

[6] Savickas M.L. (2005). The theory and practice of career construction. Career Development and Counseling: Putting Theory and Research to Work.

[7] Savickas M.L. (2011). New questions for vocational psychology: Premises, paradigms, and practices. J. Career Assess..

[8] Santisi G., Magnano P., Platania S., Ramaci T. (2018). Psychological resources, satisfaction, and career identity in the work transition: An outlook on Sicilian college students. Psychol. Res. Behav. Manag..

[9] York T.T., Gibson C., Rankin S. (2015). Defining and measuring academic success. Pract. Assess. Res. Eval..

[10] Friedlander L.J., Reid G.J., Shupak N., Cribbie R. (2007). Social support, self-esteem, and stress as predictors of adjustment to university among first-year undergraduates. J. Coll. Stud. Develop..

[11] Ajzen I., Fishbein M. (2000). Attitudes and the attitude-behavior relation: Reasoned and automatic processes. Eur. Rev. Soc. Psychol..

[12] Goodman S., Jaffer T., Keresztesi M., Mamdani F., Mokgatle D., Musariri M., Schlechter A. (2011). An Investigation of the Relationship between Students’ Motivation and Academic Performance as Mediated by Effort. S. Afr. J. Psychiatry.

[13] Richardson M., Abraham C., Bond R. (2012). Psychological correlates of university students’ academic performance: A systematic review and meta-analysis. Psychol. Bull..

[14] Boerchi D., Magnano P., Lodi E. (2021). The high school competencies scale (H-Comp Scale): A First Validation Study. Eur. J. Investig. Health Psychol. Educ..

[15] Creed P.A., Patton W., Prideaux L.A. (2007). Predicting change over time in career planning and career exploration for high school students. J. Adolesc..

[16] Gushue G.V., Scanlan K.R.L., Pantzer K.M., Clarke C.P. (2006). The relationship of career decision-making self-efficacy, vocational identity, and career exploration behavior in African American high school students. J. Career Dev..

[17] Caprara G.V., Vecchione M., Alessandri G., Gerbino M., Barbaranelli C. (2011). The contribution of personality traits and self-efficacy beliefs to academic achievement: A longitudinal study. Br. J. Educ. Psychol..

[18] Bankston C.L., Zhou M. (2002). Being well vs. doing well: Self-esteem and school performance among immigrant and non-immigrant racial and ethnic groups. Int. Migrat. Rev..

[19] Lockett C.T., Harrell J.P. (2003). Racial identity, self-esteem, and academic achievement: Too much interpretation, too little supporting data. J. Black Psychol..

[20] Schmidt J.A., Padilla B. (2003). Self-esteem and family challenge: An investigation of their effects on achievement. J. Youth Adolesc..

[21] Verkuyten M., Brug P. (2002). Ethnic identity achievement, self-esteem, and discrimination among Surinamese adolescents in the Netherlands. J. Black Psychol..

[22] Wong M.S.W., Watkins D. (2001). Self-esteem and ability grouping: A Hong Kong investigation of the big fish little pond effect. Educ. Psychol..

[23] Creed P.A., Patton W., Bartrum D. (2002). Multidimensional properties of LOT-R: Effects of optimism and pessimism on career and well-being related variables in adolescents. J. Career Assess..

[24] Kenny M.E., Walsh-Blair L.Y., Blustein D.L., Bempechat J., Seltzer J. (2010). Achievement motivation among urban adolescents: Work hope, autonomy support, and achievement-related beliefs. J. Vocat. Behav..

[25] Diemer M.A., Blustein D.L. (2007). Vocational hope and vocational identity: Urban adolescents’ career development. J. Career Assess..

[26] Juntunen C.L., Wettersten K.B. (2006). Work hope: Development and initial validation of a measure. J. Couns. Psychol..

[27] Craparo G., Magnano P., Paolillo A., Costantino V. (2018). The Subjective Risk Intelligence scale. The development of a new scale to measure a new construct. Curr. Psychol..

[28] Nicholson N., West M. (1988). Managerial Job Change: Men and Women in Transition.

[29] Evans D., Roeser S., Hillerbrand R., Sandin P., Peterson M. (2012). Risk Intelligence. Handbook of Risk Theory.

[30] Apgar D. (2006). Risk Intelligence: Learning to Manage What We Don’t Know.

[31] Funston F., Wagner S. (2010). Surviving and Thriving in Uncertainty: Creating the Risk Intelligent Enterprise.

[32] Shalley C.E.K., Zhou J., Oldham G.R. (2004). The effects of personal and contextual characteristicson creativity: Where should we go from here?. J. Manag..

[33] Zammitti A., Russo A., Santisi G., Magnano P. (2021). Personal Values in Relation to Risk Intelligence: Evidence from a Multi-Mediation Model. Behav. Sci..

[34] Lent R.W., Singley D., Sheu H., Schmidt J.A., Schmidt L.C. (2007). Relation of social-cognitive factors to academic satisfaction in engineering students. J. Career Assess..

[35] Kumar S.P.K., Dileep P. (2006). Academic Life Satisfaction Scale and Its Effectiveness in Predicting Academic Success.

[36] Lodi E., Boerchi D., Magnano P., Patrizi P. (2017). College Satisfaction Scales (CSS): The mediating role of contextual satisfaction on the relationship between self-efficacy and general life satisfaction. Appl. Psychol. Bull..

[37] Negricea C.I., Edu T., Avram E.M. (2014). Establishing influence of specific academic quality on student satisfaction. Proc. Soc. Behav. Sci..

[38] Lent R.W., Taveira M., Sheu U.B., Singley H.D. (2009). Social cognitive predictors of academic adjustment and life satisfaction in Portuguese college students: A longitudinal analysis. J. Vocat. Behav..

[39] Lent R.W., Singley D., Sheu U.B., Gainor K.A., Brenner B.R., Treistman D., Ades L. (2005). Social cognitive predictors of domain and life satisfaction: Exploring the theoretical precursors of subjective wellbeing. J. Couns. Psychol..

[40] Schmitt N., Oswald F.L., Friede A., Imus A., Merritt S. (2008). Perceived fit with an academic environment: Attitudinal and behavioral outcomes. J. Vocat. Behav..

[41] Tracey T.J., Robbins S.B. (2006). The interest–major congruence and college success relation: A longitudinal study. J. Vocat. Behav..

[42] Nauta M.M. (2007). Assessing college students’ satisfaction with their academic majors. J. Career Assess..

[43] Nurmi J.E., Aunola K., Salmela-Aro K., Lindroos M. (2003). The role of success expectation and task-avoidance in academic performance and satisfaction: Three studies on antecedents, consequences and correlates. Contemp. Educ. Psychol..

[44] Cabras C., Mondo M. (2018). Future orientation as a mediator between career adaptability and life satisfaction in university students. J. Career Dev..

[45] Boileau L., Gaudreau P., Gareau A., Chamandy M. (2021). Some days are more satisfying than others: A daily-diary study on optimism, pessimism, coping, and academic satisfaction. Br. J. Educ. Psychol..

[46] Rezaei A., Khosroshahi J.B. (2018). Optimism, social intelligence and positive affect as predictors of university students’ life satisfaction. Eur. J. Ment. Health.

[47] Yuan L.M., Zhang R.S., Zhao H.C., Liu H.C. (2006). A research on the relationship between optimism and psychological well-being. Chin. J. Clin. Psychol..

[48] Magnano P., Lodi E., Zammitti A., Patrizi P. (2021). Courage, Career Adaptability and Readiness as Resources to Improve Well-Being during the University-to-Work Transition in Italy. Int. J. Environ. Res. Public Health.

[49] Blanch A., Aluja A. (2010). Job involvement in a career transition from university to employment. Learn. Individ. Differ..

[50] Presti A.L., Capone V., Aversano A., Akkermans J. (2021). Career Competencies and Career Success: On the Roles of Employability Activities and Academic Satisfaction during the School-to-Work Transition. J. Career Dev..

[51] Vela J.C., Lerma E., Whittenberg J.F., Hinojosa Y., Rodriguez K. (2019). The Role of Positive Psychology, Cultural, and Family Factors in Latina/o College Students’ Vocational Outcome Expectations. J. Employ. Couns..

[52] Marques S.C., Lopez S.J., Pais-Ribeiro J.L. (2011). “Building Hope for the Future”: A Program to Foster Strengths in Middle-School Students. J. Happiness Stud..

[53] Larsen D.J., Stege R. (2012). Client accounts of hope in early counseling sessions: A qualitative study. J. Couns. Dev..

[54] Folkmann M.N. (2010). Enabling creativity. Imagination in design processes. First International Conference on Design Creativity.

[55] The Jamovi Project (2021). Jamovi (Version 1.6). https://www.jamovi.org.

[56] Diener E., Wirtz D., Tov W., Kim-Prieto C., Choi D., Oishi S., Biswas-Diener R. (2012). New well-being measures: Short scales to assess flourishing and positive and negative feelings. Soc. Indic. Res..

[57] Seligman M.E.P. (2012). Flourish: A Visionary New Understanding of Happiness and Well-Being.

[58] Lent R.W., Brown S.D. (2008). Social Cognitive Career Theory and Subjective Well-Being in the Context of Work. J. Career Assess..

[59] Snyder C.R., Harris C., Anderson J.R., Holleran S.A., Irving L.M., Sigmon S.T., Yoshinobu L., Gibb J., Langelle C., Harney P. (1991). The will and the ways: Development and validation of an individual-differences measure of hope. J. Personal. Soc. Psychol..

[60] Lent R.W., Brown S.D., Hackett G. (1994). Toward a unifying social cognitive theory of career and academic interest, choice, and performance. J. Vocat. Behav..

[61] Lent R.W. (2008). Understanding and promoting work satisfaction: An integrative view. Handbook of Counseling Psychology.

[62] Bandura A. (1986). Social Foundations of Thought and Action: A Social Cognitive Theory.

[63] Inchausti F., MacBeth A., Hasson-Ohayon I., Dimaggio G. (2020). Psychological Intervention and COVID-19: What We Know So Far and What We Can Do. J. Contemp. Psychother..

